# Lateralization and Distalization Shoulder Angles in Reverse Shoulder Arthroplasty: Are They Still Reliable and Accurate in All Patients and for All Prosthetic Designs?

**DOI:** 10.3390/jcm14041393

**Published:** 2025-02-19

**Authors:** Koray Şahin, Hakan Batuhan Kaya, Christos Koukos, Mehmet Kapıcıoğlu, Kerem Bilsel

**Affiliations:** 1 Department of Orthopedics and Traumatology, Bezmialem Vakif University, 34093 Istanbul, Turkey; parisabatu@gmail.com (H.B.K.); kapicioglum@gmail.com (M.K.); 2 Sports Trauma and Pain Institute, 54655 Thessaloniki, Greece; koukos_christos@hotmail.com; 3 Department of Orthopedics and Traumatology, Acibadem University Fulya Hospital, 34349 Istanbul, Turkey; kbilsel@gmail.com

**Keywords:** reverse shoulder arthroplasty, lateralization shoulder angle, distalization shoulder angle, critical shoulder angle, acromial index

## Abstract

**Background:** Recently, the lateralization shoulder angle (LSA) and distalization shoulder angle (DSA) have been proposed to assess lateralization and distalization in reverse shoulder arthroplasty (RSA). However, there is insufficient evidence about the influence of patient anatomy and prosthesis design on these measurements. This study aims to investigate the impact of patient anatomy and implant design on LSA and DSA measurements and to assess the validity of the previously reported “optimal” ranges for these parameters. **Methods:** Patients who underwent the RSA procedure using four different prosthetic designs between April 2014 and June 2023 were retrospectively evaluated. Postoperative LSA and DSA measurements were compared according to implant design, preoperative glenoid morphology (Favard classification), and the Hamada grade. The correlation of LSA and DSA with preoperative shoulder anatomy (critical shoulder angle, CSA, and acromial index, AI) was also assessed. **Results:** In total, 135 shoulders were included in the study, with a mean age of 71.7 ± 7.9 years. The mean LSA was 88.4 ± 11.8° and mean DSA was 40.6 ± 12.5°. According to prosthetic design, both mean LSA and DSA values differed significantly (*p* < 0.05). Lateralized designs (Groups I and IV) had significantly higher mean LSA values. The Favard classification and Hamada grade of shoulders did not show a significant influence on LSA and DSA measurements (*p* > 0.05). DSA was observed to be significantly correlated with CSA and AI (*p* < 0.05; r = −0.27 and −0.189, respectively). **Conclusions:** Prosthetic design and preoperative shoulder anatomy had a significant influence on LSA and DSA measurements in RSA. Optimal LSA and DSA values may lack validity and reliability and should not be applied to all patients.

## 1. Introduction

In recent years, reverse shoulder arthroplasty (RSA) has become a popular procedure for the treatment of various shoulder pathologies with expanding indications [1]. The initial design by Paul Grammont [2], which consisted of an inlay humeral cup with a high neck-shaft angle (NSA) of 155° and a glenosphere without lateral offset, made a breakthrough in RSA history since previous designs were considered elusive for the treatment of cuff tear arthropathy (CTA). Grammont’s design offered the medialization of the center of rotation and distalization of the humerus relative to the acromion and thus aimed to obtain increased deltoid muscle tension and, subsequently, higher degrees of shoulder abduction and elevation. Even though satisfying long-term clinical outcomes and high survival rates have been reported [3,4], the medialized Grammont design has been attributed to an inability to restore active external rotation and to high rates of scapular notching [5,6]. This has led to the development of the “lateralization” concept to obtain less medialization and less distalization of RSA in order to overcome these issues [5,6,7,8]. Design modifications have been made either on the humeral side with the use of inlay humeral cups, on humeral stems with more anatomic NSAs of 135° to 145° [6,7], or on the glenoid side with the use of metallic augments [8] or bony increased-offset (BIO) RSA [5], to achieve a lateralization effect. The lateralization of the center of rotation offered a better postoperative rotational range of motion [9] and a lower risk of scapular notching [4,10].

Determining the optimum amount of lateralization and distalization in RSA has been one of the fundamental questions studied. Recently, a common method to evaluate lateralization and distalization was introduced by Boutsiadis et al. [11], which proposed the “lateralization shoulder angle“ (LSA) and “distalization shoulder angle” (DSA). The authors reported a significant correlation between these radiological measurements and clinical outcomes and also offered optimum value intervals of 75–95° and 40–65° for LSA and DSA, respectively. Even though the effectiveness and high reproducibility of these two angular measurements have been confirmed by some studies [12], other studies reported opposing findings with no or negligible effect of LSA and DSA on clinical outcomes [13,14,15]. In addition, there is no sufficient evidence to support preoperative shoulder anatomy and the design of the prosthesis and how these influence LSA and DSA measurements.

The purpose of the present study is to assess the influence of preoperative patient anatomy and different implant designs on final LSA and DSA measurements and to evaluate the validity of previously reported “optimum” ranges for these different parameters. Our hypothesis was that the use of different prosthesis designs and patient anatomies would have a significant influence on postoperative LSA and DSA values, and previously reported “optimum” ranges would not be valid in all conditions.

## 2. Materials and Methods

### 2.1. Study Design and Setting

This retrospective comparative study was conducted in a tertiary university hospital clinic, which is a referral center for shoulder disorders. Medical records, including demographic and surgical data of all patients who underwent RSA surgery between April 2014 and June 2023, were reviewed. Institutional review board approval from a relevant local board was obtained (IRB ID: 170133; date: 30 October 2024)) by the initiation of the study, and all included patients provided informed consent about the use of their medical record data for the purpose of publication.

### 2.2. Patient Selection

Inclusion criteria were as follows: (1) primary RSA for CTA; (2) the availability of preoperative and postoperative true anteroposterior (AP) shoulder radiographs and shoulder computed tomography (CT) scans; and (3) the availability of surgical data on used implants. Exclusion criteria were determined as follows: (1) the revision of RSA, (2) RSA for other indications such as acute proximal humeral fracture, fracture sequelae, glenohumeral osteoarthritis, humeral head avascular necrosis, infection sequelae, and failed arthroscopic rotator cuff repair or tumors; (3) a history of any previous surgical intervention to the affected shoulder; and (4) lateralization using the BIO-RSA technique.

### 2.3. Surgical Procedure

All interventions were performed by the senior author, who is an experienced, fellowship-trained shoulder surgeon. All procedures were performed under general anesthesia combined with interscalene nerve block with patients positioned in the beach-chair position. The deltopectoral approach was preferred in all patients. Subscapularis tenotomy was performed using the peel-off technique, and the tendon was repaired whenever possible. Since this study included solely primary RSA cases, uncemented humeral stems were used in all patients. A standard surgical technique and instrumentation were performed, respecting the technical instructions of the manufacturing companies. No metallic augments were used in order to obtain glenoid-sided lateralization in any patient. All glenospheres were standard hemispheres with no augmented lateral offset.

During the study period, 4 different prostheses were used, which constituted study groups according to the prosthesis design: Group I comprised the Biomet Comprehensive Shoulder System (Biomet, Warsaw, IN, USA), which is a highly lateralized RSA design using inlay humeral cup and an NSA of 147°. Group II comprised Lima SMR Reverse (LimaCorporate, Udine, Italy), which is a medialized design with an inlay humeral cup and an NSA of 150°. Group III comprised Delta Xtend (DePuy Orthopaedics, Warsaw, IN, USA), which is a medialized design based on the original Grammont design with an inlay humeral cup and an NSA of 155°. Finally, Group IV comprised Next Shoulder Solutions (Next, Ankara, Turkey), which is also a highly lateralized design with an inlay humeral cup and 135° of NSA.

### 2.4. Radiologic Assessment and Study Groups

Radiologic assessments were performed using preoperative and postoperative true AP shoulder radiographs of the included patients. All radiological measurements were performed by a single author who was blinded to patient data using RadiAnt DICOM Viewer Software (Version 2024.1, 64.bit, Medixant, Poznań, Poland). No specific software for preoperative planning was used.

The critical shoulder angle (CSA) and the acromial index (AI) were measured on preoperative radiographs in order to assess preoperative shoulder anatomy. CSA was defined as the angle between the line connecting the superior and inferior poles of the glenoid and the line extending from the inferior pole of the glenoid to the most lateral edge of the acromion. AI was determined as the ratio of the distance from the plane of the glenoid cavity to the lateral edge of the acromion and the distance from the plane of the glenoid cavity to the lateral edge of the greater tuberosity. Secondly, coronal plane glenoid morphology was assessed using Favard’s classification [16], which was described as follows: E0: no glenoid erosion, E1: central concentric glenoid erosion, E2: superior eccentric erosion limited to superior pole, E3: global superior eccentric erosion, and E4: inferior erosion. On preoperative images, cuff tear arthropathy was also graded using the Hamada classification [17]: grade I: acromiohumeral interval ≥ 6 mm, grade II: acromiohumeral interval ≤ 5 mm, grade III: acetabulization of acromion, grade IV: glenohumeral osteoarthritis, and grade V: humeral head collapse.

**Figure 1:**
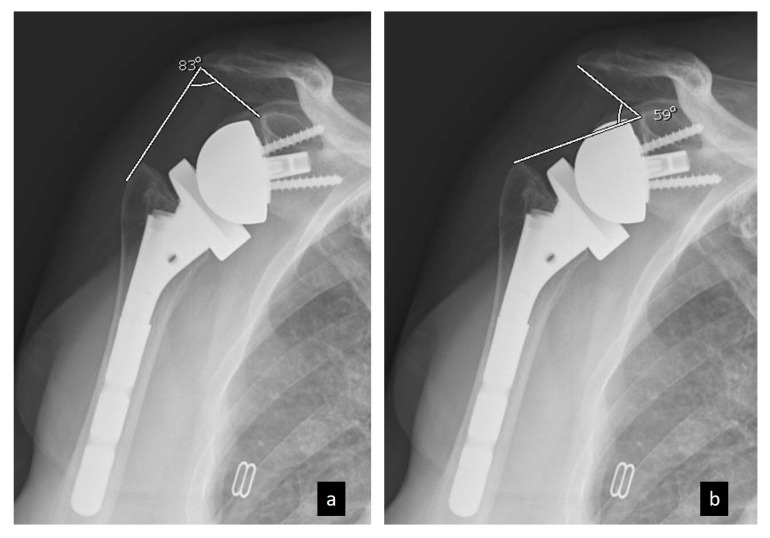
Radiological measurements of (**a**) lateralization shoulder angle (LSA) and (**b**) distalization shoulder angle (DSA) on a true anteroposterior shoulder radiograph.

On postoperative radiograph images, LSA and DSA were measured following the method described by Boutsiadis et al. [11]. LSA was defined as the angle formed between the line connecting the superior glenoid tubercle to the most lateral border of the acromion and the line extending from the most lateral edge of the acromion to the most lateral edge of the greater tuberosity (Figure 1). DSA was defined as the angle formed between the line extending from the most lateral edge of the acromion to the superior glenoid tubercle and the line extending from the superior glenoid tubercle to the most superior edge of the greater tuberosity (Figure 1).

### 2.5. Statistical Analysis

The mean, median, standard deviation, range, and percentage were the statistical methods used in order to analyze the study data. The Gaussian distribution of continuous variables was tested using the Shapiro–Wilk test, Kolmogorov–Smirnov test, and histograms. Analysis of variance was used in order to compare mean differences in LSA and DSA according to prosthesis design, glenoid morphology, and CTA level with Tukey’s test for post hoc multiple pairwise comparisons. The comparison of categorical data was performed using the chi-square test. The relationship between preoperative and postoperative angular measurements was examined using Pearson’s correlation test and scatter plots. All statistical analyses were performed using GraphPad Prism Software for Windows (Version 9.3.0, San Diego, CA, USA), and the significance level was set at *p* = 0.05.

## 3. Results

During the study period, 389 RSA procedures were performed for 374 patients (15 patients underwent sequentially bilateral surgery), and 135 shoulders (of 120 patients) who met the inclusion criteria were included in this study. The mean age of patients was 71.7 ± 7.9 years. There were 23 male (19.2%) and 97 female (80.8%) patients. According to postoperative radiologic measurements, the mean LSA value (88.4 ± 11.8°) was within the optimum limits described by Boutsiadis et al. [11] (75–95°). In total, 77 (57.0%) shoulders had postoperative LSA values within the optimum interval. However, the mean DSA value was 40.6 ± 12.5°, which was almost at the inferior limit of the optimum interval (40–65°). Moreover, only 60 (44.4%) shoulders had DSA values within the optimum range.

**Figure 2:**
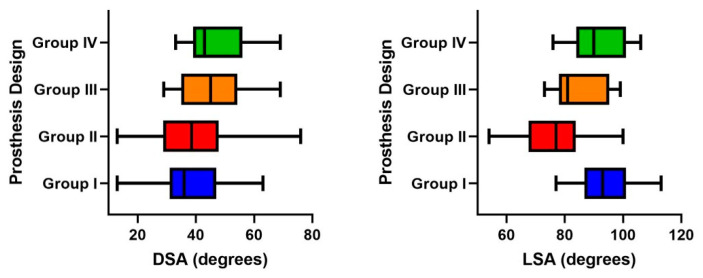
Graphs illustrating distribution of mean and range values for distalization and lateralization shoulder angles in each study group. (DSA: distalization shoulder angle; LSA: lateralization shoulder angle).

Biomet Comprehensive (Group I—inlay, 147°) prosthesis was implanted in 70 (51.9%) shoulders, Lima SMR (Group II—inlay, 150°) prosthesis was implanted in 28 (20.7%) shoulders, Depuy Delta Xtend (Group III—inlay, 155°) prosthesis was implanted in 18 (13.3%) shoulders, and Next Shoulder Solutions (Group IV—inlay, 135°) prosthesis was implanted in 19 (14.1%) shoulders. The comparison of mean LSA and DSA values between different prosthesis designs showed significant differences (*p* < 0.0001 and *p* = 0.01, respectively) (Figure 2). Post hoc pairwise comparisons (Supplementary Materials) revealed that lateralized designs (Group I and IV) had significantly higher mean LSA values compared to medialized designs (Group II and III), and Group II (Lima SMR Reverse) had the lowest mean LSA value of 76.3 ± 11.3°. For DSA, mean values were outside the optimum range in groups I and II (37.9 ± 11.2° and 39.9 ± 14.9°). However, the number of shoulders resulting within the optimum range of LSA and DSA were comparable between four prosthesis designs (*p* = 0.74 and *p* = 0.08) (Table 1).

**Figure 3:**
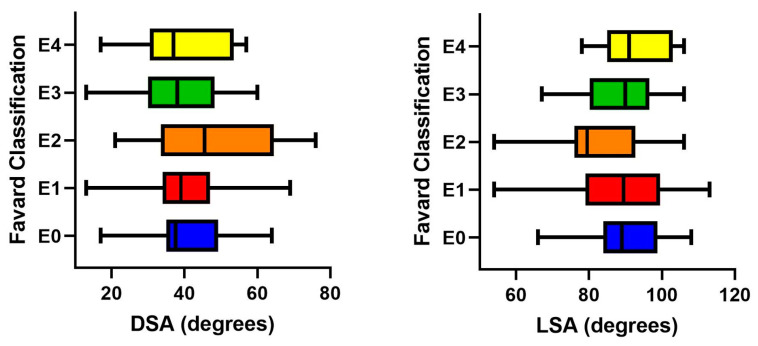
Graph illustrating the distribution of mean and range values for distalization and lateralization shoulder angles according to glenoid morphology (Favard classification). The glenoid morphology did not have a significant impact on both angular measurements, and mean values were comparable between different morphology groups. (DSA: distalization shoulder angle; LSA: lateralization shoulder angle).

The preoperative glenoid morphology of patients according to Favard’s classification [16] was classified as E0 in 36 (26.7%) patients, E1 in 46 (34.1%) patients, E2 in 16 (11.9%) patients, E3 in 28 (20.7%) patients, and as E4 in 9 (6.7%) patients. Glenoid morphology did not seem to have an influence on LSA and DSA measurements, and the mean LSA and DSA values were comparable between different glenoid morphology groups (*p* = 0.21 and *p* = 0.13, respectively) (Figure 3). Similarly, the rate of shoulders that had LSA and DSA measurements within optimum ranges did not have a significant difference between glenoid morphology groups (*p* = 0.49 and *p* = 1.00, respectively) (Table 2).

**Figure 4:**
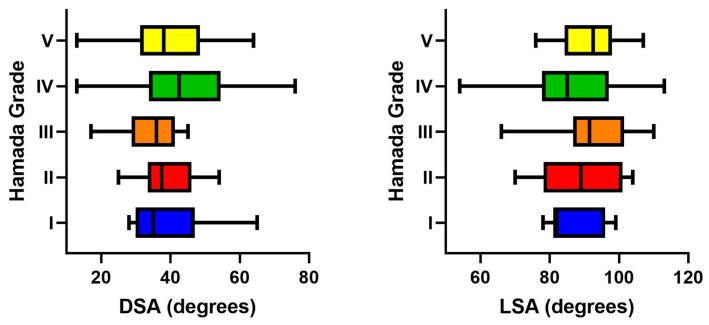
Graph illustrating the distribution of mean and range values for distalization and lateralization shoulder angles according to the Hamada grade. The Hamada grade did not have a significant impact on both angular measurements, and mean values were comparable between different grades. (DSA: distalization shoulder angle; LSA: lateralization shoulder angle).

According to the Hamada classification, there were 11 (8.1%) grade I patients, 24 (17.8%) grade II patients, 18 (13.3%) grade III patients, 62 (45.9%) grade IV patients, and 20 (14.8%) grade V patients. The mean values of LSA and DSA were comparable between different Hamada grades (*p* = 0.29 and *p* = 0.07, respectively) (Figure 4). Accordingly, the rate of shoulders that ended up with LSA and DSA measurements within the optimum ranges did not differ significantly between different Hamada grades (*p* = 0.65 and *p* = 0.71, respectively) (Table 3).

**Figure 5:**
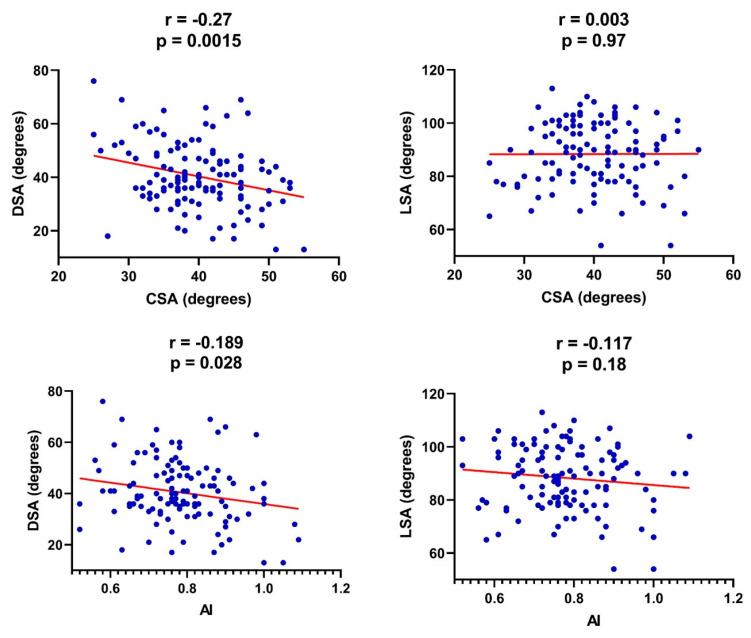
Scatter plot graphs illustrating correlation between preoperative shoulder morphology (critical shoulder angle and acromial index) and postoperative angular measurements. (DSA: distalization shoulder angle; LSA: lateralization shoulder angle; CSA: critical shoulder angle; AI: acromial index).

DSA and LSA were significantly and negatively correlated (*p* < 0.0001, r = −0.355). No significant correlation was observed between LSA and CSA (*p* = 0.97, r = 0.003) and AI (*p* = 0.18, r = −0.117). However, there was a significant negative correlation between DSA and CSA (*p* = 0.002, r = −0.27) and AI (*p* = 0.03, r = −0.189) (Figure 5).

## 4. Discussion

The main finding of the present study is that the design of the prosthesis had a significant influence on postoperative angular measurements, especially LSA values, which were found to be significantly higher when a lateralized RSA design was used. Secondly, some aspects of preoperative shoulder anatomy showed an important impact on angular measurements. Preoperative glenoid morphology (the Favard classification) and Hamada grade did not have a significant relationship either with LSA or DSA. However, CSA and AI showed a significant negative correlation with DSA. These findings imply that optimal LSA and DSA values might not be valid and reliable for every scenario and should not be generalized to all patients.

In 2018, Boutsiadis et al. [11] described a simple and reproducible method, LSA and DSA, in order to evaluate the amount of lateralization and distalization of RSA in CTA patients. The authors indicated that regardless of the surgical technique used, LSA and DSA were significantly correlated with functional outcomes and ranges of motion. They also defined the following optimal ranges: a DSA value of 40–65° was reported to be associated with the highest active elevation, and an LSA value of 75–95° was reported to be associated with the highest external rotation. However, in the present study, approximately only half of the patients (57.0% for LSA and 44.4% for DSA) had angular measurements inside these optimal ranges. Moreover, the mean DSA value of our cohort was 40.6 ± 12.5°, which was almost at the inferior limit of the optimum range. Similar findings have also been reported by previous reports [14], which indicated inconsistent LSA and DSA results compared to previously described optimum ranges. These findings indicate that the existence of optimal LSA or DSA values should be questioned [18].

A subsequent study by Erickson et al. [12] partially supported the findings of Boutsiadis et al. [11], and reported that only LSA was significantly associated with functional outcomes, but this association was observed for limited changes. On the contrary, the majority of recent reports suggest opposing findings, indicating no association of LSA and DSA with clinical outcomes [13,14,15]. However, most of these mentioned studies still outlined LSA and DSA as reproducible measurements with high intra- and interobserver reliability [15,19].

In a recent study conducted with 51 patients, Marsalli et al. [18], also evaluated the influence of implant design on LSA and DSA measurements. Three different (two lateralized and one medialized design) RSA implant designs (FH Arrow^TM^ system, Biomet Comprehensive^TM^ shoulder system, and Mathys Affinis^TM^ reverse system) were tested. According to our findings, they reported significantly higher LSA values in lateralized designs compared to medialized designs. However, unlike our results, the mean LSA and DSA values for all three implant designs were within the optimum range in their study; thus, the authors concluded that adequate LSA and DSA could be achieved regardless of the RSA design used. On the contrary, in our study, the mean DSA value was outside of the optimum range in Groups I and II (37.9° and 39.9°, respectively); the mean LSA value was almost at the most inferior limit of the range (76.3°) in Group II. Moreover, in our study, the rate of patients who had LSA and DSA values within the optimum range was considerably lower compared to previous findings. However, the mean values for LSA and DSA were quite similar in both studies for whole study populations (88.4° vs. 88.5° for LSA and 40.6° vs. 45.2° for DSA) despite the difference in the rate of patients inside the optimum ranges. This difference may be due to variations during radiological measurements or due to the different implants used. Considering these findings, we think that the implication which was made by Marsalli et al. [18] should be reconsidered, and further evidence should be sought to reach a conclusion. It is evident that defining exact optimum limits for a parameter that is so much affected by various factors, such as implant design and patient anatomy, is not reliable.

For a radiologic measurement method to be precise and ideal, it should be minimally influenced by other factors. Another important point for LSA and DSA is that these measurements share the same anatomical landmarks as CSA and AI, which means that patient anatomy can have a significant impact on LSA and DSA measurements. A recent study [20] showed that both LSA and DSA had a significant correlation with CSA. Accordingly, Marsalli et al. [18] also reported a significant correlation between DSA and CSA. In accordance with these findings, our results show that DSA is negatively correlated with CSA and AI. This implies that despite achieving adequate distalization, the DSA value can decrease if the patient has a high CSA, which shows low reliability and validity of these angular measurements. In a recent study by Okutan et al. [20], new measurement methods, the lateralization index (LI) and distalization index (DI), were defined using 3D planning software (Blueprint, Wright, Memphis, TN, USA) in order to overcome these aforementioned drawbacks of LSA and DSA. The authors indicated that LI and DI were significantly correlated with lateralization and distalization, and unlike angular measurements, they could provide reliable estimations. However, further research is still needed to verify this implication.

Although the findings of our study suggest that patient anatomy and RSA implant design have a significant effect on postoperative LSA and DSA values, it should be kept in mind that these angular measurements, especially DSA, do not depend solely on these variables. The present study failed to reveal a significant influence of glenoid morphology (Favard classification) or the Hamada grade on LSA and DSA values. However, there might be other unanticipated patient-related anatomical factors that may have an association with these measurements. Moreover, some surgeon-related factors such as surgical technique, the level of humeral cut, size of the glenosphere, and thickness of the polyethylene and humeral tray might also play an important role in final LSA and DSA measurements.

There are several limitations to our study that need to be noted. The retrospective nature of this study is the first limitation that needs to be mentioned, as this may have caused some possible biases. However, we tried to avoid selection bias by only including CTA patients. There might also be inevitable technical differences during procedures, such as the humeral cut level, the selection of glenosphere size, or polyethylene thickness, which could have influenced the standardization of the surgical technique and which can be considered as a limitation. However, we tried to minimize this risk by using a standard surgical technique and including only the patients who were operated on by a single surgeon. The radiologic measurements might have been affected by the shoulder position as it was not always possible to obtain an ideal true AP shoulder radiograph. However, it has been emphasized that this reflects the routine difficulty of daily clinical practice and cannot be easily eliminated [15]. Another limitation is that all radiographic measurements were performed by a single author, and no inter- or intraobserver reliability analysis were performed, which may have caused possible evaluation bias. However, LSA and DSA have already been previously attributed to high reproducibility and interobserver reliability [15]. The correlation of our findings with clinical outcomes could not be evaluated in this study due to the unavailability of clinical outcome measures, which can be considered another limitation. This should be examined in future research, and the clinical implications of the presented findings should be examined.

There are also some important strengths related to this study. The first strength that needs to be mentioned is the inclusion of a considerably larger cohort compared to most of the previous reports on this topic [11,13,15,19,20]; thus, we could avoid the possible bias caused by small cohort size. Secondly, all included patients were operated on by a single author in a single center using a standard surgical technique, which provided a homogeneous cohort, unlike previous reports. However, conducting a single-center study also has some potential drawbacks that cannot be ignored, such as limited generalizability, institutional and selection bias, and lack of variability in surgical or rehabilitation techniques. Patient characteristics, surgical or rehabilitation techniques, and follow-up protocols may differ widely across institutions and may limit how applicable the findings are to other centers. Another strength of our study is the assessment of four different implant designs, unlike other studies, which confined their evaluations to less numerous designs. And also, to our knowledge, this is the first study to evaluate the effect of preoperative glenoid morphology and Hamada grade on LSA and DSA measurements, which can be considered another strength of the present study.

## Figures and Tables

**Table 1:** Mean LSA and DSA measurements in different prosthesis designs and rate of shoulders within the optimum range for LSA and DSA.

	Group I (*n* = 70)	Group II (*n* = 28)	Group III (*n* = 18)	Group IV (*n* = 19)	*p* Value
LSA°	93.3 ± 9.2°	76.3 ± 11.3°	84.6 ± 9.0°	91.6 ± 9.9	**<0.0001 ^a^**
LSA 75–95° n (%)	40 (57.1)	14 (50.0)	12 (66.7)	11 (57.9)	0.74 ^b^
DSA°	37.9 ± 11.2°	39.9 ± 14.9°	45.7 ± 11.0°	46.9 ± 12.0°	**0.01 ^a^**
DSA 40–65° n (%)	26 (37.1)	11 (39.3)	12 (66.7)	11 (57.9)	0.08 ^b^

Data are presented as the mean ± standard deviation or frequency (percentage). LSA: lateralization shoulder angle, DSA: distalization shoulder angle, ^a^: one-way ANOVA test, ^b^: chi-square test. Bold *p* values indicate statistical significance.

**Table 2:** Mean LSA and DSA measurements in different preoperative glenoid morphology groups according to the Favard classification and rate of shoulders within the optimum range for LSA and DSA.

	E0 (*n* = 36)	E1 (*n* = 46)	E2 (*n* = 16)	E3 (*n* = 28)	E4 (*n* = 9)	*p* Value
LSA°	89.1 ± 10.6°	89.0 ± 13.0°	82.3 ± 13.6°	88.5 ± 10.2°	92.8 ± 10.1°	0.21 ^a^
LSA 75–95° n (%)	19 (52.8)	23 (50.0)	11 (68.8)	19 (67.9)	5 (55.6)	0.49 ^b^
DSA°	40.3 ± 10.8°	40.1 ± 11.6°	48.1 ± 16.4°	37.9 ± 12.6°	39.9 ± 13.7°	0.13 ^a^
DSA 40–65° n (%)	16 (44.4)	21 (45.7)	7 (43.8%)	12 (42.9)	4 (44.4)	1.00 ^b^

Data are presented as the mean ± standard deviation or frequency (percentage). LSA: lateralization shoulder angle, DSA: distalization shoulder angle, ^a^: one-way ANOVA test, ^b^: chi-square test.

**Table 3:** Mean LSA and DSA measurements according to different Hamada grades and rates of shoulders within the optimum range for LSA and DSA.

	Grade I (*n* = 11)	Grade II (*n* = 24)	Grade III (*n* = 18)	Grade IV (*n* = 62)	Grade V (*n* = 20)	*p* Value
LSA°	87.6 ± 8.2°	89.1 ± 11.9°	91.3 ± 12.1°	86.3 ± 12.9°	91.9 ± 8.6°	0.29 ^a^
LSA 75–95° n (%)	8 (72.7)	11 (45.8)	10 (55.6)	36 (58.1)	12 (60.0)	0.65 ^b^
DSA°	40.3 ± 11.6°	38.8 ± 8.3°	34.3 ± 8.7°	43.5 ± 13.9°	39.8 ± 14.1	0.07 ^a^
DSA 40–65° n (%)	5 (45.5)	9 (37.5)	6 (33.3)	31 (50.0)	9 (45.0)	0.71 ^b^

Data are presented as the mean ± standard deviation or frequency (percentage). LSA: lateralization shoulder angle, DSA: distalization shoulder angle, ^a^: one-way ANOVA test, ^b^: chi-square test.

## Data Availability

The raw data supporting the conclusions of this article will be made available by the authors upon request.

## Supplementary Materials

The following supporting information can be downloaded at: https://www.mdpi.com/article/10.3390/jcm14041393/s1, Results of post-hoc pairwise comparison analysis for lateralization shoulder angle (LSA) and distalization shoulder angle (DSA) between prosthesis design groups.

## Abbreviations

The following abbreviations are used in this manuscript:

LSA	Lateralization shoulder angle
DSA	Distalization shoulder angle
RSA	Reverse shoulder arthroplasty
CSA	Critical shoulder angle
AI	Acromial index
NSA	Neck-shaft angle
CTA	Cuff tear arthropathy
BIO-RSA	Bony increased-offset reverse shoulder arthroplasty
