# Supplementary material for: Lateralization and Distalization Shoulder Angles in Reverse Shoulder Arthroplasty: Are They Still Reliable and Accurate in All Patients and for All Prosthetic Designs?
Source: J Clin Med. 2025 Feb 19;14(4):1393. doi: 10.3390/jcm14041393 (PMC11856366; doi:10.3390/jcm14041393)
Supplement: Supplementary file 1 [file jcm-14-01393-s001.zip › jcm-3480242-supplementary.pdf]

**Group comparison**	**Mean difference**	**95% CI of difference**	**p value**
Group I vs Group II	17.1	11.4 to 22.7	**<0.0001**
Group I vs Group III	8.69	1.99 to 15.4	**0.005**
Group I vs Group IV	1.67	-4.89 to 8.22	0.91
Group II vs Group III	-8.36	-16.0 to -0.707	**0.026**
Group II vs Group IV	-15.4	-22.9 to -7.85	**<0.0001**
Group III vs Group IV	-7.02	-15.4 to 1.31	0.13

Results of post-hoc pairwise comparison analysis for lateralization shoulder angle (LSA) between prosthesis design groups. (CI: confidence interval. Bolded p values indicate statistical significance)

**Group comparison**	**Mean difference**	**95% CI of difference**	**p value**
Group I vs Group II	-2.01	-9.08 to 5.05	0.88
Group I vs Group III	-7.75	-16.1 to 0.601	0.079
Group I vs Group IV	-8.98	-17.2 to -0.803	**0.025**
Group II vs Group III	-5.74	-15.3 to 3.81	0.402
Group II vs Group IV	-6.97	-16.4 to 2.43	0.22
Group III vs Group IV	-1.23	-11.6 to 9.17	0.99

Results of post-hoc pairwise comparison analysis for distalization shoulder angle (DSA) between prosthesis design groups. (CI: confidence interval. Bolded p values indicate statistical significance)
